# Management of Locally Advanced Prostate Cancer: A Scoping Review of Contemporary Evidence and Emerging Approaches

**DOI:** 10.7759/cureus.89863

**Published:** 2025-08-12

**Authors:** Mayowa Adefehinti, Althea O George, Obinna Enemoh, Blessing Adenipekun, Mobolaji Akinwale, Abiodun Akintayo, Quadri A Sanni, Ajibola A Adebisi, Ehizokhae Erewele, Oluwabukunmi Onipede, Ojeyemi Oore-ofe, Toluwanimi P Ibikunle, Opemipo O Ajani

**Affiliations:** 1 Urology, Peterborough City Hospital, Peterborough, GBR; 2 Urology, The Royal London Hospital, London, GBR; 3 General Surgery, Chesterfield Royal Hospital, Chesterfield, GBR; 4 Surgery, William Harvey Hospital, Kent, GBR; 5 Surgery, University Hospital Southampton NHS Foundation Trust, Southampton, GBR; 6 Urology, South Warwickshire University NHS Foundation Trust, West Midlands, GBR; 7 General Surgery, Epsom and St.Helier University NHS Foundation Trust, London, GBR; 8 Surgery, Olabisi Urgent Care Hospital, Lagos, NGA; 9 Urology, University of Ilorin, Ilorin, NGA; 10 General Practice, Lagoon Hospital, Lagos, NGA; 11 Pediatrics, Atlantis Pediatrics Hospital, Lagos, NGA; 12 Pubic Health, London Metropolitan University, London, GBR

**Keywords:** advanced locally prostate cancer – lapc, androgen deprivation therapies, apalutamide, multimodal treatments, precision oncology, psma pet, radical prostatectomy, radiotherapy, scoping review

## Abstract

Locally advanced prostate cancers (LAPC) are a clinical dilemma due to their biological heterogeneity and multiple algorithmic treatment options. Recent years have seen a great deal of progress in management, both in established methods and new modalities. This had led to a necessity to systematically map out existing evidence. This scoping review intends to systematically integrate and summarize the zeitgeist research in the area of the care of LAPC to advance the available knowledge, discuss emergent management strategies, and identify evidence needs in different healthcare settings.

A scoping review was undertaken following the Joanna Briggs Institute methodology and reported following the Preferred Reporting Items for Systematic reviews and Meta-Analyses extension for Scoping Reviews (PRISMA-ScR) guidelines. A primary study search was conducted on six electronic databases (MEDLINE, Embase, Scopus, Web of Science, CINAHL Plus, and Cochrane Library) and gray literature sources published between 2013 and April 2025. The Population-Concept-Context (PCC) framework guided the eligibility. Data were plotted and thematically synthesized into six key domains: hormonal therapy, radiotherapy innovations, surgical strategies, new systemic therapies, imaging improvements, and real-life evidence.

Six studies were included, consisting of a randomized controlled trial, an observational study, and a diagnostic review. The main themes were the benefits of multimodal treatment, the impact of prostate-specific membrane antigen (PSMA) positron emission tomography (PET) imaging and genomic profiling, and the role of second-generation androgen receptor inhibitors and regional disparities in access to treatments. Combination therapies showed oncologic benefits, but raised concerns about patients’ quality-of-life outcomes.

The management of LAPC is moving to a related precision-based, multimodal paradigm. Although current knowledge supports more aggressive and individualized therapy, there are still data gaps in long-term outcomes, global uptake, and patient-reported measures. Future research needs to be based on inclusive longitudinal studies that span between clinical innovation and real-world application.

## Introduction and background

Prostate cancer is one of the most common malignancies in men and a leading cause of morbidity and mortality from cancer [1]. Although a large number of prostate cancers are diagnosed in the early stages with an indolent course, a significant proportion of patients present with locally advanced prostate cancer (LAPC). LAPC is defined as extra-prostatic extension (T3-T4), involvement of the seminal vesicles or regional lymph nodes without distant metastases (M0) [2]. Given its heterogeneous biological behavior and different treatment outcomes, this disease stage presents a distinct therapeutic dilemma. Therefore, a management strategy that addresses the dichotomy between disease control and quality of life is required.

The incidence of LAPC has regional variations, which are mostly due to screening availability, diagnostic practices, and hospital infrastructure [3]. In high-income countries, stage migration through the introduction of prostate-specific antigen (PSA) testing was observed with a relative LAPC cases [3]. Due to PSA screening becoming more conservative owing to the fears of overdiagnosis, there is increasing evidence of an increase in LAPC cases in recent years [4]. However, in low- and middle-income countries (LMICs), delayed diagnoses because of limited resources, and LAPC still represents a common presentation [5]. A thorough understanding of new approaches to this disease stage in different clinical settings and geographical regions is needed to address its global burden. LAPC refers to tumors that have extended beyond the prostate capsule but have not metastasized to distant organs.

The management of LAPC has an inherent complexity in the form of an interplay of several modalities, such as radical prostatectomy, external beam radiotherapy (EBRT), androgen deprivation therapy (ADT), and, sometimes, brachytherapy or chemotherapy, among others [6]. ADT is a systemic treatment used to reduce or block the production or action of androgens (male hormones, primarily testosterone), which fuel the growth of prostate cancer cells. These treatments may be used alone or in combination in patients with different tumor characteristics, comorbidities, and clinical judgment. Although combination therapies have been shown to improve oncologic outcomes, these therapies are also associated with an increased risk of adverse outcomes such as urinary incontinence, bowel dysfunction, and sexual health problems, which compromise quality of life [2]. In this way, clinicians are becoming more burdened with the need to weigh the benefits of aggressive treatment against the risk of long-term damage.

The recent developments in imaging, molecular profiling, and targeted therapy are changing the face of prostate cancer treatment. Developments such as prostate-specific membrane antigen (PSMA) PET imaging and genomic classifiers are improving risk stratification and tailoring of treatment plans, especially in ambiguous or borderline LAPC cases [7]. Moreover, second-generation androgen receptor inhibitors and immunotherapeutic agents, among others, are being investigated for their applicability in the locally advanced context, although historically, they have been used for metastatic disease [8]. Such developments highlight the necessity for continued assessment of both established and emerging modalities of intervention.

Given the ever-changing nature of treatment strategies for LAPC and the huge amount of clinical data emerging, there is an urgent need to summarize existing knowledge in a comprehensive and organized way. Although numerous narrative and systematic reviews exist, most of them address the early-stage or metastatic prostate cancer, while very few are devoted to the locally advanced stage only. Furthermore, current reviews tend to focus on individual modalities or outcomes, rather than examining the range of interventions and patient experiences of the full spectrum of available interventions.

Therefore, this scoping review aims to map the breadth of available literature on the management of LAPC, with a particular focus on contemporary evidence and emerging therapeutic approaches. By employing a scoping methodology, this review will identify key concepts, gaps in the literature, and future directions for research and clinical practice. 

This work is intended to give a basic reference for clinicians, researchers, and policy makers involved in prostate cancer care to enable evidence-based decision-making and to identify areas that need further investigation.

Research questions

This scoping review is guided by three central research questions. (1) What are the current treatment strategies for LAPC, and how are they being implemented across different clinical and geographical contexts? (2) What emerging modalities and innovations are being explored in the management of LAPC? (3) Where do gaps exist in terms of clinical evidence, treatment outcomes, and patient-centered care?

## Review

RPThis scoping review was undertaken based on the methodology framework offered by Arksey and O’Malley [9], adapted through the Joanna Briggs Institute (JBI) guidelines and the Preferred Reporting Items for Systematic reviews and Meta-Analyses extension for Scoping Reviews (PRISMA-ScR) [10]. The review protocol was conducted before the search strategy adoption, though it was not placed on an open repository because of institutional rules.

A risk-of-bias assessment was not conducted in this scoping review, as per the methodological guidance provided by the JBI and the PRISMA-ScR reporting guidelines. The primary objective of a scoping review is to map the breadth and nature of evidence on a given topic, rather than to evaluate the quality or synthesize results from individual studies. As such, assessing the methodological quality or risk of bias of included studies is not a mandatory component. This approach allows for the inclusion of a wide range of evidence, irrespective of methodological rigor, which is consistent with the exploratory nature of scoping reviews. No formal statistical analysis or meta-analysis was conducted, as the purpose was to map the breadth of existing literature rather than to assess effect sizes.

Eligibility criteria

This scoping review includes studies that examine the management of LAPC in adult males (aged 18 years and older), specifically those with clinical stage T3-T4, N0-N1, M0 disease. Studies focusing exclusively on early-stage or metastatic (M1) prostate cancer were excluded.

Included studies described clinical interventions or care pathways, treatment outcomes (either oncologic or quality-of-life-related), or explored novel and emerging therapeutic approaches. Eligible studies were those that provided insight into the clinical management of LAPC, including modalities such as radiotherapy, surgery, androgen deprivation therapy (ADT), and innovative treatments.

To ensure contemporary relevance, only peer-reviewed primary research published in English between January 2013 and April 2025 was included. This encompassed randomized controlled trials (RCTs), observational studies (e.g., cohort studies), qualitative research, and mixed-methods studies. Studies not addressing treatment strategies such as those focused solely on epidemiology, screening, or diagnostic techniques, were excluded.

Additionally, the review excluded editorials, commentaries, letters to the editor, conference abstracts without complete data, study protocols, and reviews. Only studies conducted in any healthcare setting and across all geographic regions were considered, provided they met the other inclusion criteria.

PCC framework justification

This review utilized the Population-Concept-Context (PCC) scoping review framework to guide the eligibility criteria and to focus the evidence mapping [11].

Population: Adult males with LAPC

Concept: Clinical management strategies, including surgical, radiotherapeutic, systemic, and emerging approaches

Context: All healthcare settings, across both high-income and low- and middle-income countries (LMICs)

Information sources and search strategy

A thorough search strategy was developed with the help of an academic librarian. The electronic databases searched included: MEDLINE (through PubMed), Embase, CINAHL Plus, Web of Science, and Scopus. Other grey literature was found through Google Scholar and institutional repositories. Search terms were created with the use of controlled vocabulary (e.g., MeSH) and keywords about LAPC and its management (e.g., “locally advanced prostate cancer”, “radiotherapy,” “androgen deprivation therapy”, “PSMA”, “novel therapies”). Boolean operators, truncations, and filters were used to increase sensitivity. 

Filters

Filters applied during the literature search included studies published in English, from 2013 to 2025, involving human subjects. An example of the search string in MEDLINE is presented in Appendix A.

The last search was held in April 2025. All search results were exported to EndNote for de-duplication and screening.

Selection of Sources of Evidence

After de-duplication, all titles and abstracts were screened independently by two reviewers. Full-text screening was performed on all these studies considered potentially eligible. Discrepancies were resolved through discussion or by consultation with a third reviewer.

**Figure 1:**
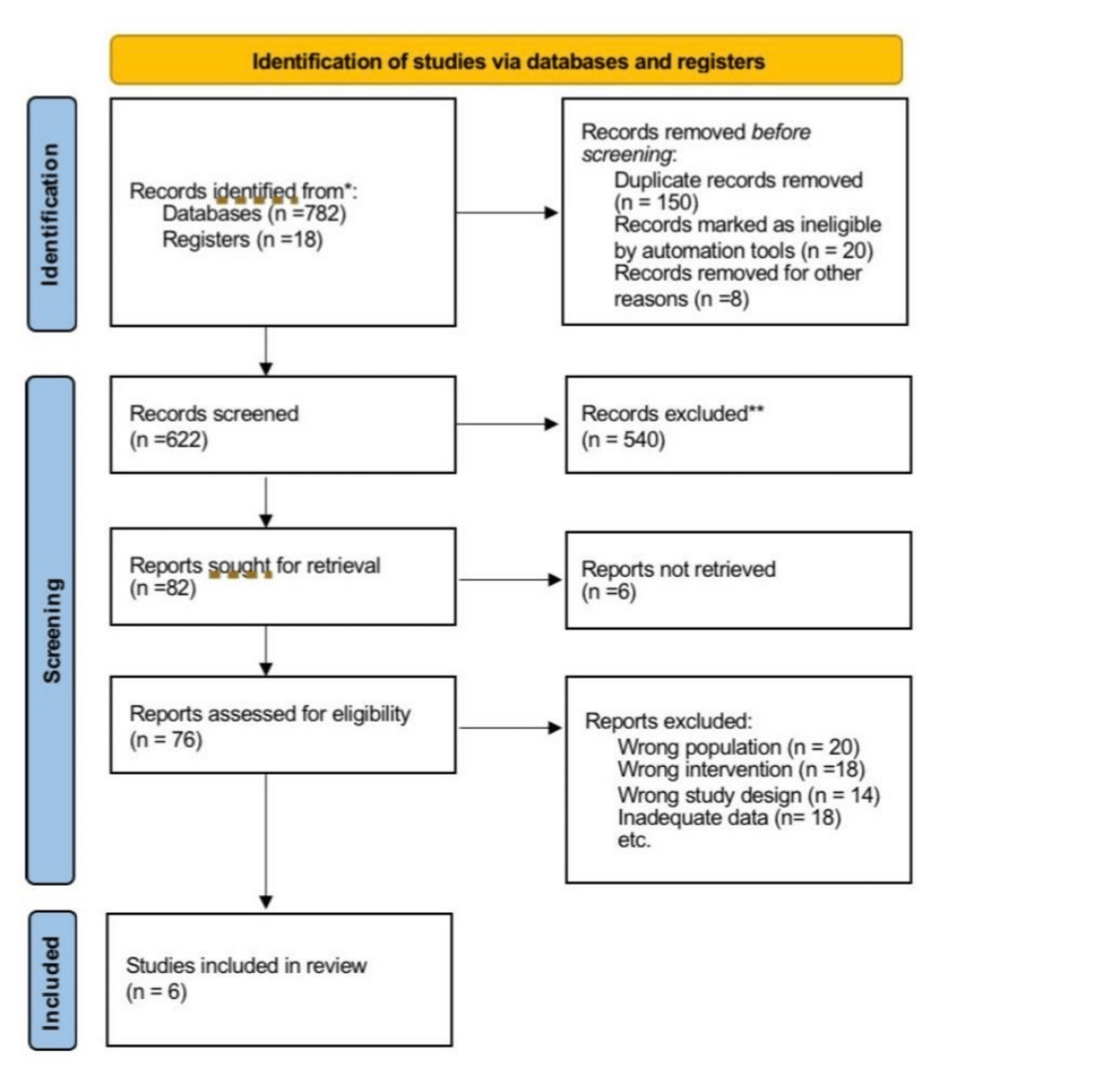
PRISMA flowchart. PRISMA, Preferred Reporting Items for Systematic reviews and Meta-Analyses

The selection process is described by the PRISMA-ScR flowchart (Figure 1), and the number of records identified, screened, excluded, and included is provided, as well as reasons for exclusion at the full-text stage.

Data Charting Process

A standardized data extraction form was developed to guide the charting process. The following key items were extracted from each included study: citation details (author(s) and year), country of study, study design (e.g., RCT, cohort, case-control, qualitative), sample size and participant characteristics, study setting (e.g., hospital, cancer center, outpatient facility), type of intervention(s) examined, comparator(s) where applicable, reported outcomes (e.g., survival rates, recurrence, treatment-related toxicity, and quality of life), key findings, and any stated gaps or recommendations for future research.

Two reviewers extracted data from the included studies separately. Inconsistencies were confirmed through discussion and consensus. The extracted data were summarized in tables to facilitate thematic synthesis (Appendix B).

Synthesis of Results

This scoping review took a descriptive and thematic synthesis approach. The extracted data were grouped based on the major intervention categories such as radiotherapy, surgery, systemic therapy, and novel treatments. Subthemes were developed using iterative reading and coding of the findings from the study. We did not perform a formal risk of bias assessment since the major objective of this review was to map the gamut of available evidence, not to decide about the quality or efficacy of individual studies [12].

Each theme generated a narrative summary with frequency count of study type, location, and outcome, if applicable. Furthermore, the changing trends in the clinical management approaches were identified.

PRISMA-ScR Compliance

This review follows the PRISMA-ScR checklist (Appendix C) in order to provide transparency and completeness. The selection and inclusion process, as per best practice standards, is demonstrated in the PRISMA-ScR flow diagram (Figure 1).

Results

Study Selection

A comprehensive literature search yielded 800 records (782 from databases and 18 from trial registers) as shown in the PRISMA-ScR diagram (Figure 1). After removing 178 records due to duplication or automation ineligibility, 622 studies remained for title and abstract screening. Of these, 82 full-text articles were assessed for eligibility. After further exclusions, 6 studies met all inclusion criteria and were retained for final synthesis.

To ensure rigor and reduce selection bias, two reviewers independently screened titles and abstracts, and any disagreements were resolved through discussion or consultation with a third reviewer. This approach aligns with the JBI scoping review methodology and ensures transparency and reliability in study selection [12].

The process of study selection is presented in the PRISMA-ScR flow diagram (Figure 1).

Thematic Analysis of Included Studies

The six final studies were grouped thematically by their primary research focus. These studies spanned diverse methodologies, including RCTs, observational studies, and diagnostic evaluations (Table 1).

**Table 1:** Thematic analysis of the included studies. LAPC, locally advanced prostate cancer

Theme	Code	Definition	Associated Studies
Systemic Treatment - Hormonal/Chemotherapy	T1	Use of androgen deprivation therapy (ADT) and systemic therapies (e.g., docetaxel, apalutamide) in managing LAPC	James et al. (2016) [13]
Radiotherapy Advances	T2	Innovations in dose scheduling, particularly ultra-hypofractionation	Widmark et al. (2019) [14]
Surgical Management	T3	Comparative evaluation of radical prostatectomy versus radiotherapy	Stranne et al. (2018) [15]
Emerging Therapies and Biomarkers	T4	The efficacy of the combination of apalutamide and standard ADT	Shore et al. (2024) [16]
Diagnostic Imaging	T5	Use of PSMA PET for staging and therapy planning	Houshmand et al. (2023) [17]
Real-World Practice and Implementation	T6	Observational data on treatment variability and adherence to guidelines	Freedland et al. (2023) [18]

To structure the analysis, the PICO (Population, Intervention, Comparison, Outcome) framework was applied.

Theme 1: Hormonal therapy: Hormonal therapy is still a mainstay of therapy for LAPC, and its purpose is to reduce androgen levels and thereby suppress tumour progression. The most influential study in this area is the STAMPEDE trial by James et al. [13], which assessed the effect of the addition of docetaxel as a chemotherapeutic drug to the standard ADT. Carried out at various sites, the trial involved men with high-risk, non-metastatic and metastatic prostate cancer, which provided powerful information regarding systemic cancer treatment strategies. The results showed significant improvement in overall survival and progression-free survival with docetaxel and ADT vs. ADT alone. Since then, these outcomes have redefined the standard of care by encouraging early integration of chemotherapy in eligible LAPC patients. Critically, the trial demonstrated that early intensification of treatment leads to better outcomes, favoring a move to combination approaches rather than sequential treatments in high-risk prostate cancer.

Theme 2: Radiotherapy innovations: Radiotherapy has seen great development in recent years, especially in fractionation techniques, which are designed to produce optimal doses whilst reducing the accommodation of the patient and side effects. A landmark study in this area is the hypofractionated radiotherapy for prostate cancer (HYPO-RT-PC) trial performed by Widmark et al. [14], a phase 3, non-inferiority RCT in men with intermediate- to high-risk prostate cancer, including a subset of men with locally advanced disease. The study compared ultra-hypofractionated (UHF) radiotherapy, in which the total dose of 42.7 Gy was spread over just seven sessions over two and a half weeks, to conventional fractionation of 78.0 Gy in 39 fractions over eight weeks. Results showed that the UHF radiotherapy schedule was non-inferior to failure-free survival at five years without a significant increase in late toxicity. Most significantly, such an approach provides practical benefits in the form of shortened duration of therapy, increased compliance of patients, and rational usage of resources in actual clinical settings. Based on these observations, it is possible to integrate UHF radiotherapy into the treatment algorithm for LAPC in cases where it is required due to either limitations of the health care infrastructure or patient preference, respectively.

Theme 3: Surgical management: Surgical intervention, especially radical prostatectomy (RP), is now being considered more achievable in the management of LAPC, especially when it is incorporated into a multimodal approach. The SPCG-15 (Scandinavian Prostate Cancer Group Study Number 15) trial, a prospective randomized controlled study by Stranne et al. [15], contributes valuable comparative information between RP and radiotherapy with ADT. This trial included patients with LAPC and sought to establish whether surgery could offer the same or better oncological outcomes in the long term. Preliminary outcomes showed that the two treatment arms similarly achieved cancer control, mostly in biochemical recurrence and metastasis-free survival. Nonetheless, adverse event profiles were notably different: RP was more often linked with urinary incontinence and sexual dysfunction, while patients treated by radiotherapy experienced more cases of digestive tract side effects, i.e., bowel urgency, and rectal bleeding. These findings are useful in emphasizing the value of shared decision-making, in which clinicians individualize treatment plans based on patient-specific factors such as age, comorbid diseases, baseline function, and patients’ personal value of quality of life.

Moreover, with increasing global adoption of robotic-assisted prostatectomy and evidence suggesting lower blood loss and shorter hospital stays, future reviews may further investigate the comparative efficacy of open, laparoscopic, and robot-assisted surgical approaches in LAPC [19]. However, such comparisons were not the focus of the SPCG-15 trial and remain underrepresented in current LAPC literature.

Although outside the scope of this review’s inclusion criteria, early-phase studies on stereotactic radiotherapy and PARP inhibitors (e.g., olaparib for BRCA1/2 mutations) are emerging and could potentially expand the therapeutic landscape for LAPC [20]. These approaches warrant separate systematic exploration as more clinical outcome data become available.

Theme 4: Emerging therapies and biomarkers: The recent development of systemic therapies and molecular diagnostics has created novel opportunities for the treatment of LAPC in the neoadjuvant setting. The efficacy of the combination of apalutamide (an androgen receptor inhibitor) and standard ADT in patients with high-risk localized or locally advanced disease candidates for RP was tested in the PROTEUS trial, as reported by Shore et al. [16]. Apalutamide has shown proven activity in castration resistant situations, and hence this trial aimed at testing its efficacy in earlier stages of the disease. The findings showed that the addition of apalutamide substantially increased pathologic complete response (pCR) rates and reduced residual tumor volume at surgery compared with ADT alone. These results are supportive of the performance of intensified neoadjuvant therapy to enhance surgical outcomes and reduce recurrence risk. In addition, the trial demonstrates the growing reliance on individualized systemic treatment before definitive local therapy. Future investigations may consider the long-term survival effects, as well as incorporating genomic biomarkers to inform patient selection for such enhanced approaches.

Theme 5: Advanced imaging techniques: Staging accuracy in the treatment of LAPC plays a vital role in treatment planning, and equally, in the prognosis. CT and bone scans are known to have limitations in detecting Micro metastatic disease; thus, traditional imaging modalities are essential. This was answered by the Avidity trial conducted by Houshmand et al., who studied the clinical relevance of PSMA PET/CT in patients with high risk or LAPC. This proposed trial was designed to determine if PSMA PET/CT provided more sensitivity and specificity in the detection of nodal and distal metastases than standard imaging [17]. Early results indicated that PSMA PET/CT was able to identify metastatic lesions not visible on routine scans, which made significant changes in treatment choices in many cases. These changes ranged from ramping up systemic therapy to the addition of targeted radiotherapy to oligometastatic sites or reclassification to non-surgical candidates. The trial supports the importance of sophisticated imaging as a means of increasing precision medicine and personalization of therapeutic methods in LAPC.

Theme 6: Real-world evidence: Although clinical trials offer controlled environments to determine the effectiveness of treatment, real-world evidence contributes invaluable insights into the performance of therapies in routine clinical practice. Freedland et al. [18] reported a US-based observational study that studied treatment patterns and outcomes of patients with localized and LAPC. Using a large national registry, the study collected information on patient demographics, desired interventions, timelines of treatment, and survival outcomes. The analysis showed high heterogeneity in the use of treatment modalities, including the use of ADT, surgery, and radiotherapy, which differed from region to region and health care settings. Interestingly, the outcome was different based on the combination of treatment and the type of facility used, which raises concerns about the consistency of care. The study stressed the need for the development and dissemination of such evidence-based, standardized treatment protocols to decrease this variability and increase patient outcomes. Further, it underlined the utility of actual world data in highlighting gaps between clinical trial and practice, informing future policy and studies in LAPC management.

Critical Appraisal Using CASP

The methodology quality of the included studies was evaluated using the Critical Appraisal Skills Programme (CASP) tool. Each of the six studies was appraised using the relevant CASP checklists according to their design (RCTs or observational studies). The appraisal revealed the following:

Study design: All studies had clearly defined research questions and employed appropriate methodologies

Validity: Randomization and blinding were adequately addressed in the RCTs, reducing the risk of bias.

Results: The studies provided detailed and reliable results, with appropriate statistical analyses.

Applicability: Findings were relevant to clinical practice, although some studies had limitations regarding generalizability due to specific patient populations or healthcare settings.

**Table 2:** Critical appraisal using CASP. CASP, Critical Appraisal Skills Programme; RCT, randomized controlled trial

Study	Design	Clear aim?	Appropriate methodology?	Recruitment strategy clearly described?	Bias addressed?	Results clearly reported?	Relevant to practice?	CASP quality rating
James et al. (2016) [13]	RCT	Yes	Yes	Yes	Yes	Yes	Yes	High
Widmark et al. (2019) [14]	RCT	Yes	Yes	Yes	Yes	Yes	Yes	High
Stranne et al. (2018) [15]	RCT	Yes	Yes	Yes	Yes	Yes	Yes	High
Shore et al. (2024) [16]	RCT	Yes	Yes	Yes	Somewhat	Yes	Yes	Moderate-high
Houshmand et al. (2023) [17]	Diagnostic Review	Yes	Yes	Yes	Somewhat	Yes	Yes	Moderate-high
Freedland et al. (2023) [18]	Observational Cohort	Yes	Yes	Yes	Limited	Yes	Yes	Moderate

Discussion 

The purpose of this scoping review was to synthesize current evidence and emerging strategies for the management of LAPC. This review has emphasized the progression from conventional monotherapies towards precision-guided, multi-modal approaches, through the inclusion of six high-quality studies in systemic, locoregional, diagnostic, and observational arenas. The findings capture the maturity of certain practices, such as ADT and increasing clinical utility of novel hormonal agents, sophisticated imaging modalities, and real-world implementation data.

Summary of Key Findings

Throughout all themes, one of the most recurrent messages is the promotion of treatment intensification and personalization. The STAMPEDE (Systemic Therapy in Advancing or Metastatic Prostate Cancer: Evaluation of Drug Efficacy) trial [13] confirmed the importance of early integration with ADT, while Shore et al. [16] delivered essential evidence of what apalutamide in the post-RP setting in patients with high-risk LAPC means. Their study revealed that apalutamide delayed disease relapse significantly, had better PSA-based results, and therefore should be considered as adjuvant systemic therapy.

Strategies for radiotherapy are also being changed. Widmark et al. [14] study, HYPO-RT-PC trial, showed that UHF radiotherapy is not only feasible but also non-inferior as far as failure-free survival is concerned. This gives clinicians a more convenient option that is shorter in duration while maintaining efficacy with the same as well as reduced resource demands and patient burden.

Surgical management is still an important mode of treatment for LAPC. The primary outcomes of the SPCG-15 trial conducted by Stranne et al. [15] revealed similar oncological outcomes between primary RP and the combination of radiotherapy and ADT. Nevertheless, the side effect profiles of these approaches were unique: Greater urinary incontinence was linked to surgery, while radiotherapy tended to have a higher number of bowel complications. These findings confirm the principle of shared decision-making and underline the need to individualize therapy according to patient comorbidities, preferences, and quality of life considerations.

Houshmand et al. [17] took this a step higher in terms of the role of precision medicine in this context by documenting the clinical implications of PSMA PET imaging in the LAPC staging and treatment planning. Their review demonstrated that the application of PSMA PET greatly enhances the detection of lesions relative to conventional imaging, enabling more precise staging, change in treatment, and possible earlier intervention. Additionally, Freedland et al. [18] added real-world evidence that described substantial differences in treatment patterns and highlighted the existing gap between clinical trial evidence and standard practice.

Implications for Clinical Practice

These results have immediate implications for how clinicians treat LAPC now. First, there is a highly coherent experience that supports an early combination treatment approach with a special emphasis on systemic intensification using chemo-hormonal or novel hormonal agents to delay disease progression and decrease metastasis risk. The increasing evidence of adjuvant therapies such as apalutamide after prostatectomy [16] indicates a novel role for these agents even without overt metastasis.

Second, improvements in the delivery of radiotherapy and imaging are contributing to more accurate and patient-friendly procedures. Ultra-hypofractionation has similar efficacy but in a more condensed schedule, and PSMA PET is increasingly regarded as a superior alternative for initial staging and recurrence detection [21]. These innovations will probably replace legacy imaging protocols and make the treatment pathway even more personalized.

Third, there is a compelling reason to match practice to individual patient preferences, given that different modalities may have very different side effect profiles. Information from Stranne et al. [15] will provide the ground for a more perceptive discussion on long-term functional outcomes when counselling patients on surgery versus radiotherapy.

Ultimately, the diversity found in Freedland et al.’s [18] real-world study highlights the necessity for more explicit national and international guidance with consistent application in healthcare systems.

Comparison with Previous Literature

These review results correspond with the previous systematic reviews and clinical guidelines that have encouraged multimodal approaches to LAPC. Nevertheless, it brings new depth by incorporating more recent, less well-known studies focusing on key emergent areas such as PSMA-based diagnostics and new AR-targeted therapies. The newly revised data from Shore et al. [16] and Houshmand et al. [17] are representative of a movement toward personalized treatment that encompasses biomarkers and advanced imaging - dimensions that were previously underrepresented in clinical practice algorithms.

Interestingly, despite wide support from existing literature for combined modality treatment, Freedland et al. [18] show that this does not translate to the field as there is variability in how the treatment is received and utilized. This disconnect can be explained by clinician experience or local infrastructure, or by inconsistent publication of new findings from updated research. Reporting similar implementation gaps exists worldwide, and closing them will be necessary for equitable care delivery.

Gaps in Evidence and Research Priorities

Various gaps were identified in the review. First, long-term outcome data on new agents like apalutamide in earlier-stage disease are still limited. Although positive early biochemical results were reported by Shore et al. [16], further strong survival figures as well as quality-of-life evaluations are necessary to substantiate their role in adjuvant protocols.

Second, studies like SPCG-15 have mostly targeted European cohorts, which could restrict generalization of findings among diverse global populations [22]. Future research designs need to account for racial and ethnic disparities in prostate cancer occurrence and treatment outcomes better.

Third, although the diagnostic superiority of PSMA PET is now well supported [17]. There is still a need for more data on how imaging-driven shifts in staging impact survival or toxicity in practice. Further to this, cost efficiency and access inequities should be discussed before the popular use, especially in public health systems.

Finally, although patient-reported outcomes are starting to emerge in trials, the challenge of consistent inclusion and standardization persists [23]. Lack of validated quality of life data, particularly in real-world cohorts, hampers clinicians’ ability to advise patients on the tradeoff between survival benefit and treatment burden.

Strengths and Limitations of the Review

One key strength of this scoping review is its approach, which is systematic in nature and is guided by PRISMA-ScR guidelines, thematic analysis, organizational structure of PICO, and PICO-centered quality assessment using CASP. The blend of two RCTs and real-world studies brings together an encompassing picture of control strategies around LAPC management in various locations.

Nevertheless, this review has several constraints. Only English language studies were considered, which may have created language bias. Additionally, a formal meta-analysis was not performed because of the research heterogeneity in design and outcomes. This is suitable for scoping reviews, but limitations on the ability to measure effect sizes or comparative risks. Also, while only high-quality studies were chosen, new data from 2024-2025 is still becoming available, and may shortly update current conclusions.

The management of LAPC is undergoing a process of transformational change. The past decade has been littered with anecdotal evidence that favors earlier, more personalized, and multimodal interventions - increasingly under the guidance of imaging, biomarkers, and patient-reported outcomes. These advances, however, are not consistently applied, and significant evidence gaps remain, particularly concerning long-term outcomes, quality of life, and applicability across diverse populations. This review not only maps the current best practices but also prepares the ground for future inquiry to deepen and broaden the value of personalized LAPC care into all healthcare settings.

## Conclusions

This scoping review delved into the treatment of LAPC with the help of the synthesis of six high-quality studies published between 2016 and 2025, covering systemic therapy, radiotherapy, surgery, diagnostic imaging, and real-world practice. The results show an obvious shift between the traditional monotherapy approach to LAPC care towards contemporary integrated and multimodal treatment paradigms that strive for a balance between oncological control and quality of life.

Studies from large trials such as STAMPEDE and Apa-RP also support early use of systemic intensification using agents such as docetaxel and apalutamide to improve survival outcomes and recurrence. Improvements in radiotherapy, notably in the verification of UHF regimens, have practical advantages without loss of efficacy. Surgical management is still an option with the SPCG-15 trial suggesting outcomes equivalent to radiotherapy plus ADT, with completely different side effects. This increase in staging accuracy and treatment individualization was further brought about by the advent of the PSMA PET imaging, as outlined by Houshmand et al.

Although the occurrence of such developments, real-world data reveal tremendous variation in the delivery of treatment, reinforcing the need for standardized protocols and equitable access to innovations. There is still scant data on long-term outcomes, especially for newer systemic agents, and inconsistent reporting of patient-centered outcomes (e.g., quality of life, functional status).

Finally, the management of LAPC is rapidly altering, due to quality evidence and technological progress. Translation into consistent practice, however, has not been completed. Future research ought to focus on inclusivity, long-term efficacy, and integration of measures of patient-reported outcomes to ensure that the benefits of precision medicine are not only achieved but also become sustainable for all patients. This review presents a basic map of existing knowledge and highlights relevant areas for future exploration that underpin both clinical practice as well as health policy development.

## Appendices

Appendix A: Search strategy

**Table 3:** Search strategy.

Component	Search Terms	Boolean Operators
Population	"locally advanced prostate cancer" OR "LAPC" OR "high-risk prostate cancer"	OR
Intervention/Concept	"radical prostatectomy" OR "external beam radiotherapy" OR "EBRT" OR "androgen deprivation therapy" OR "ADT" OR "docetaxel" OR "apalutamide" OR "enzalutamide" OR "darolutamide"	OR
Management Context	"management" OR "treatment" OR "therapeutics"	OR
Emerging Tools/Techniques	"PSMA PET" OR "genomic classifiers" OR "imaging" OR "risk stratification"	OR
Study Type	"clinical trial" OR "observational study" OR "prospective study" OR "real-world evidence"	OR
Combined Logic	(Population) AND (Intervention/Concept) AND (Management) AND (Emerging Tools/Techniques) AND (Study Type)	AND
Limits Applied	English language, Human studies, Publication date: Jan 2018 – Apr 2025, Peer-reviewed full-text articles	AND

Appendix B: Summary table

**Table 4:** Summary table.

Author(s)	Year	Study Title / Acronym	Design	Focus	Key Findings
James et al. [13]	2016	STAMPEDE Trial	RCT	Hormonal therapy (ADT + docetaxel)	Adding docetaxel to ADT improved overall survival and progression-free survival.
Widmark et al. [14]	2019	HYPO-RT-PC	RCT	Radiotherapy – ultra-hypofractionation	Short-course radiotherapy was non-inferior to conventional treatment in terms of failure-free survival.
Stranne et al. [15]	2018	SPCG-15 Trial	RCT	Radical prostatectomy vs radiotherapy + ADT	Comparable oncologic outcomes; surgery linked to more urinary incontinence, radiotherapy to more bowel toxicity.
Shore et al. [16]	2024	Apa-RP	RCT	Apalutamide post-radical prostatectomy	Apalutamide improved PSA-based outcomes and delayed recurrence in high-risk patients.
Houshmand et al. [17]	2023	PSMA PET Imaging Review	Diagnostic Review	Advanced imaging for staging	PSMA PET improved detection accuracy and influenced treatment planning.
Freedland et al. [18]	2023	US Real-World Study	Observational	Treatment patterns and outcomes	Highlighted variability in clinical practice and the need for standardised protocols.

Appendix C: PRISMA-ScR checklist

**Table 5:** PRISMA-ScR checklist.

Section	PRISMA-ScR Checklist Item Description
Title	Identify the report as a scoping review.
Abstract	Provide a structured summary including background, objectives, eligibility criteria, sources, methods, results, and conclusions.
Introduction	Describe the rationale for the review in the context of what is already known.
Objectives	Provide an explicit statement of the questions and objectives being addressed using the PCC framework.
Eligibility Criteria	Specify characteristics of the sources of evidence used as eligibility criteria, giving rationale.
Information Sources	Describe all information sources (e.g., databases with dates of coverage) used in the search.
Search Strategy	Present the full electronic search strategy for at least one database, including any limits used.
Selection of Sources	State the process for selecting sources of evidence (i.e., screening, eligibility, inclusion).
Data Charting	Describe the methods of charting data from the included studies.
Synthesis of Results	Summarise how the results were synthesized, including themes, tables, and frequency analysis.
Results	Present the number of sources screened, assessed for eligibility, and included; give characteristics of included sources.
Discussion	Summarise the main findings, link to review objectives, limitations, and implications for future practice.
